# Unsupervised machine learning-based stratification and immune deconvolution of liver hepatocellular carcinoma

**DOI:** 10.1186/s12885-025-14242-5

**Published:** 2025-05-10

**Authors:** Mae Montserrat Reierson, Animesh Acharjee

**Affiliations:** 1 Cancer and Genomic Sciences, School of Medical Sciences, College of Medicine and Health, University of Birmingham, Birmingham, B15 2TT UK; 2 Institute of Translational Medicine, University Hospitals Birmingham NHS Foundation Trust, Birmingham, B15 2TT UK; 3 MRC Health Data Research UK (HDR), Midlands Site, UK; 4 Centre for Health Data Research, University of Birmingham, Birmingham, B15 2TT UK

**Keywords:** Unsupervised machine learning, Liver hepatocellular carcinoma, Immune deconvolution, Tumor stratification, Tumor microenvironment, Multi-modal data integration

## Abstract

**Background:**

Hepatocellular carcinoma (HCC) is the most prevalent type of liver cancer and a leading cause of cancer-related deaths globally. The tumour microenvironment (TME) influences treatment response and prognosis, yet its heterogeneity remains unclear.

**Methods:**

The unsupervised machine learning methods— agglomerative hierarchical clustering, Multi-Omics Factor Analysis with K-means++, and an autoencoder with K-means++ — stratified patients using microarray data from HCC samples. Immune deconvolution algorithms estimated the proportions of infiltrating immune cells across identified clusters.

**Results:**

Thirteen genes were found to influence HCC subtyping in both primary and validation datasets, with three genes—*TOP2A*, *DCN*, and *MT1E*—showing significant associations with survival and recurrence. *DCN*, a known tumour suppressor, was significant across datasets and associated with improved survival, potentially by modulating the TME and promoting an anti-tumour immune response.

**Conclusions:**

The discovery of the 13 conserved genes is an important step toward understanding HCC heterogeneity and the TME, potentially leading to the identification of more reliable biomarkers and therapeutic targets. We have stratified and validated the liver cancer populations. The findings suggest further research is needed to explore additional factors influencing the TME beyond gene expression, such as tumour microbiome and stromal cell interactions.

**Supplementary Information:**

The online version contains supplementary material available at 10.1186/s12885-025-14242-5.

## Introduction

Hepatocellular carcinoma (HCC) is an inflammation-associated cancer, where the tolerogenic nature of the liver continuously attempts reparations on the chronically inflamed tissue, promoting the infiltration of leukocytes and reactive oxygen species [1]. The increase in hepatocyte apoptosis and oxidative stress creates an angiogenic hypoxic microenvironment and opportunities for oncogenic DNA mutations during cell turnover [2]. Due to its often-asymptomatic development, HCC is frequently diagnosed at advanced stages, typically during routine surveillance of pre-existing chronic hepatic dysfunction using ultrasonography or detecting high serum α-fetoprotein levels (AFP) [3]. This delay in diagnosis limits the effectiveness of treatments such as curative surgical resection, liver transplantation, and ablation, leaving many patients to rely on non-curative interventions like transarterial chemoembolization and systemic therapies [4].

As the disease advances, neovascularisation in the tumour microenvironment (TME) increases resilience to therapeutic intervention by promoting immune escape, proliferation, and metastasis, underscoring the significance of early-stage diagnosis for improving prognosis and 5-year survival rates [1]. Median survival is directly correlated with tumour stage, making early detection paramount [5]. However, definitive diagnosis is reliant on medical imaging, such as multiphase CT or MRI with contrast, making it highly subjective owing to the inter- and intra-tumoral heterogeneity [6]. The Barcelona Clinic Liver Cancer (BCLC) system is the most widely used subclassification method, primarily based on Child-Pugh grade of liver dysfunction, tumour size, and AFP levels [7]. However, this approach, which focuses on the physical attributes of the lesion, does not fully account for the TME, a critical factor in accurately predicting treatment response [8].

The TME of HCC suppresses the innate tolerogenic mechanisms and creates a tolerance to the immune response and fatigue to the anti-tumour immunity, increasing in severity as the cancer advances [9]. HCC employs several immunosuppressive pathways to create a pro-tumorigenic microenvironment, such as accumulating tumour-infiltrating lymphocytes that secrete immunosuppressive cytokines, which inhibit the activation of effector T cells and natural killer cells [2]. Bahcivanci et al. [10] used immune deconvolution algorithms on microarray datasets to estimate the abundance of infiltrating immune cells and identified genes that were significant to the differentiation between HCC tumour and non-tumour samples [10]. The findings indicated *ECM1* gene as an immune-related marker through its involvement in macrophage polarisation and the observed significant differences in macrophage infiltration between tumour and non-tumour samples. This study further confirms HCC hijacks and modulates the adaptive immune response but did not look at between-patient variance. Zhang et al. (2022) employed consensus hierarchical clustering to identify four novel immune subtypes of HCC based on long-non-coding RNA [11]. Prognosis differentiated between clusters, likely due to deviating immunosuppressed TME. The studies stressed the need for more comprehensive analyses that consider the influence of TME on cancer prognosis.

The clinical variability observed in HCC suggests the presence of uncharacterised molecular subgroups, shaped in part by diverse TME compositions. Identifying these subgroups could improve patient stratification, guide personalised medicine, and uncover immune-related prognostic markers. To fully uncover the heterogeneity of HCC, powerful analytical tools that can dissect the underlying mechanisms propelling hepatocarcinogenesis need to be employed. Unsupervised machine learning (UML) is a well-established approach for elucidating hidden patterns in high-dimensional cancer datasets and subtype identification. Resende et al. (2023) applied hierarchical clustering on pre-surgical resection characteristics of HCC patients and found three subgroups based on their prognosis [12]. The UML analysis found the relationship between AFP and sex was the greatest factor behind prognosis prediction. With a similar aim but using different methodology, Niño-Ramírez et al. (2021) identified four distinct patterns of HCC patients using K-means clustering on clinical characteristics, each with a unique prognosis and survival outcome [13]. Both studies leveraged the power of UML to define homogeneous conglomerates based on multi-modal clinical features, but did not integrate immune cell profiling to explore heterogeneity at the intra-tumoural level.

**Fig. 1:**
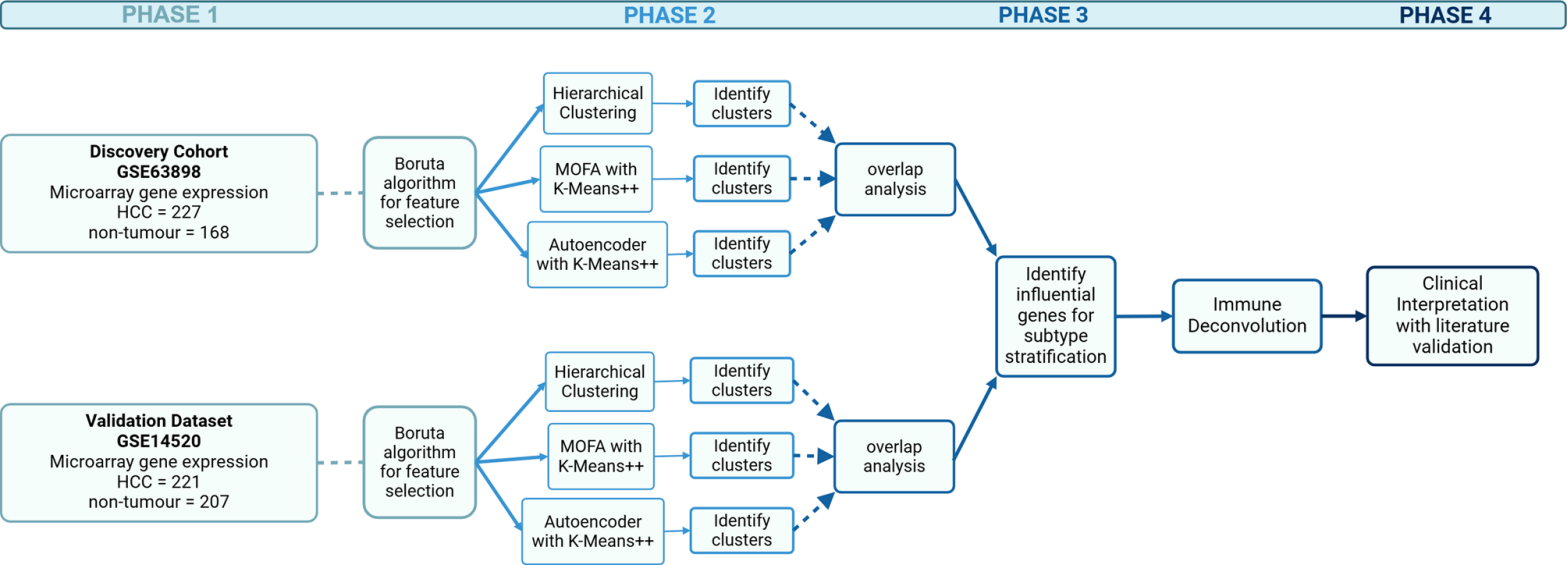
Overview of methods. Phase 1: Data discovery and pre-processing with feature selection; phase 2: subtype stratification; Phase 3: overlap analysis and gene enrichment; Phase 4: immune deconvolution and clinical interpretation. Created with BioRender.com [14]

In this study, *we* aimed to identify robust subgroups of hepatocellular carcinoma patients to explore the heterogeneity of the tumour microenvironment. *We* applied multiple unsupervised machine learning methods for robust stratification and three immune deconvolution algorithms to identify the associated immune cell abundances of these groups. *Our* research was in four phases (Fig. 1): data discovery and pre-processing with feature selection; subtype stratification; overlap analysis and gene enrichment; and immune deconvolution. The results of this study aim to provide potential immune-related gene markers as prognostic factors or therapeutic targets to promote an anti-tumour immunity.

## Materials and methods

This section outlines the methodological framework used in this study, structured according to the workflow illustrated in Fig. 1. The study is divided into four key phases. Phase 1 (Sect. “Data Discovery”–“Feature Selection”) covers data discovery, preprocessing, and feature selection using the Boruta algorithm to identify key genes. Phase 2 (Sect. “Subtype Stratification”) involves subtype stratification, where three clustering approaches—hierarchical clustering, multi-omics factor analysis (MOFA) with K-means++, and autoencoder with K-means++—are applied to identify subtypes. Phase 3 (Sect. “Overlap Analysis”–“Influential Genes and Pathway Enrichment Analysis”) focuses on overlap analysis to determine robust clusters, followed by identifying influential genes and performing pathway enrichment analysis to interpret cluster characteristics. Phase 4 (Sect. “Immune Deconvolution”) applies immune deconvolution algorithms to estimate the immune cell composition in the identified subtypes, culminating in a clinical interpretation of findings through literature validation.

### Data discovery

**Fig. 2:**
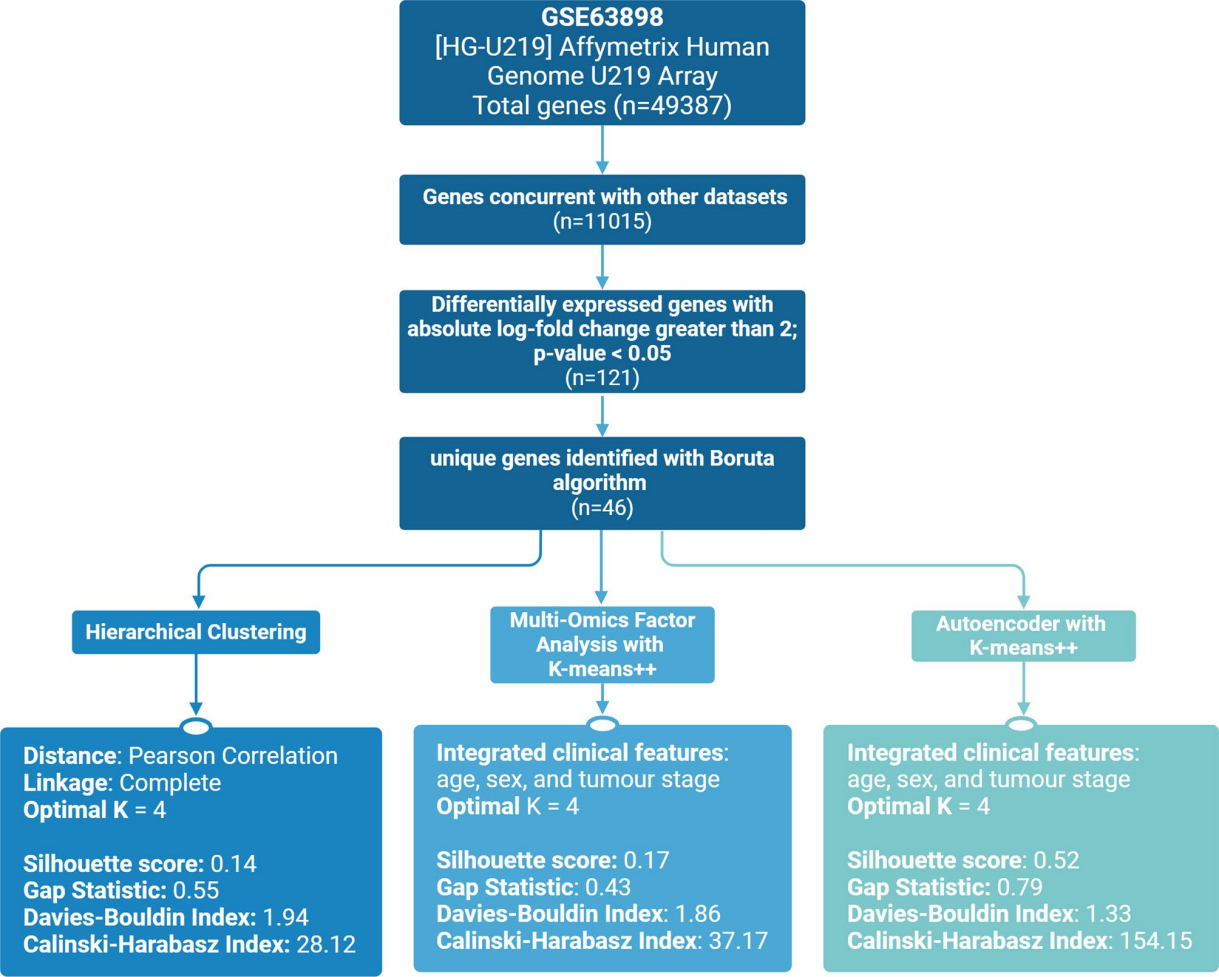
Flow diagram of data discovery process. Datasets were identified in online repositories, screened for eligibility, and included in further analysis. Datasets were excluded based on sample size, cancer progenitor, technology, or insufficient or no clinical features. Created with BioRender.com [14]

Using the electronic databases Cancer livER, Gene Expression Omnibus, and the Database of Hepatocellular Carcinoma Expression Atlas, 40 HCC gene expression datasets were screened for eligibility (Fig. 2) [15–17]. Affymetrix microarray of biotinylated coding-RNA was chosen for its comprehensive whole-genome analysis. Studies including both tumour and adjacent non-tumour samples were selected for differential gene expression analysis (DGEA) during feature selection. To capture HCC genetic heterogeneity and increase generalisability, studies with only one progenitor disease were excluded. Additionally, studies focused on HCC recurrence were excluded, as the biological mechanisms differ significantly from those in primary HCC. Clinical features were required for patient stratification, so studies with insufficient or missing clinical data were also excluded.

Two datasets meeting these criteria were identified: GSE63898 from the HEPTROMIC project (2015) was selected as the primary dataset, and GSE15420 was used for validation (Table 1) [18, 19]. Both datasets had one sample per patient that had been normalised with robust multi-array average. The primary dataset had age, sex, and BCLC tumour stage as available clinical features. One sample with missing clinical features was excluded from the primary dataset, leaving 227 tumour and 168 adjacent non-tumour samples. Thirteen samples were excluded from the validation dataset due to missing HCC samples, healthy origins, or incomplete clinical data, resulting in 221 tumour and 207 adjacent non-tumour samples.

**Table 1:** HCC discovery and validation datasets

Reference paper	GEO accession	Number of HCC samples	Number of adjacent non-tumour samples	Technology	Set
Villanueva et al., 2015 [18]	GSE63898	228	168	Affymetrix Human Genome U219 Array	Discovery
Roessler et al., 2010 [19]	GSE14520	226	220	Affymetrix HT Human Genome U133A Array	Validation

### Outlier detection

To identify potential outliers or mislabelled samples, principal component analysis (PCA) and t-distributed stochastic neighbour embedding (t-SNE) were performed on the log-transformed datasets. These dimensionality-reduction techniques work in conjunction: PCA creates uncorrelated principal components that linearly capture data variance, while t-SNE, as a non-linear method, preserves local similarities. They provide different perspectives on the data for robust outlier detection. Both techniques were implemented in R, using the *Stats* package for PCA and the *Rtsne* package for t-SNE [20, 21]. The 95% confidence intervals calculated from the Mahalanobis distances based on the approximately Chi-square distributed principal components were added to the PCA. t-SNE hyperparameters were set with a perplexity of 50 and 1000 maximum iterations to balance local and global data structure preservation. Samples were considered outliers if significantly distant from other group samples in both plots and were removed from further analysis.

### Feature selection

The gene probes from both datasets were re-annotated using *AnnotationDbi* package in R [22]. Genes lacking a corresponding HGNC name or missing in the validation dataset were excluded. To focus on only the genes specifically altered in the TME, a DGEA was performed between tumour and adjacent non-tumour samples. The *Limma* package in R uses linear models for each gene to explain its relationship to the experimental conditions. A t-statistic, moderated using an empirical Bayes method, determined if gene expression significantly differed between groups. The p-values were adjusted for multiple testing using the Benjamini-Hochberg procedure to limit the false discovery rate [23]. Genes with an adjusted p-value below 0.05 and an absolute log fold change greater than 2 were kept for further analysis.

The differentially expressed genes (DEGs) were then entered into a random forest (RF) wrapper using the *Boruta* package in R [24]. It iteratively compares the importance of each gene to a randomly permuted shadow version, eliminating those deemed less important as a method of feature selection. The RF classifier validates the importance of each gene in distinguishing between tumour and non-tumour samples, by comparing the maximum Z-score of the DEGs to the shadow attribute. 30% of the inputted DEGs tested the performance of the RF model, with the square root of the training group being used as the *mtry* hyperparameter to balance complexity and overfitting, and 1500 *trees* to capture diversity in the feature subsets. A grid search method was used for hyperparameter tuning through10-fold cross validation with 10 repeats.

### Subtype stratification

Phase 2 (Fig. 1) has been broken into sub-Sect. “Hierarchical Clustering” to “K-means ++” to describe the UML methods used for patient stratification.

Three unsupervised clustering methods were selected for their complementary strengths: hierarchical clustering for its ability to identify nested patterns within the data, making it effective for detecting hierarchical subgroup structures; MOFA for its capacity to integrate structured and unstructured data, enabling the extraction of biologically relevant latent factors; and autoencoders to capture complex, non-linear relationships by compressing high-dimensional features into a lower-dimensional representation. K-means + + was used for downstream clustering in MOFA and autoencoder models due to its improved centroid initialisation, which enhances convergence stability compared to standard K-means [34].Hotelling T-Squared analysis assessed the distribution of the clinical features using the *Hotelling* package in R [25]. This test compares the mean vectors of the clinical features of one cluster against all others in a holistic analysis of the multivariate data. If a cluster was found to have a statistically different composition of clinical features, a two-sided student’s T-test with a 0.95 confidence level was performed to determine how the cluster differed significantly from the rest of the study population - this was performed using the *t.test()* function in base R [20].

#### Hierarchical clustering

Agglomerative hierarchical clustering was performed using the *ConsensusClusterPlus* package in R, resampling 80% of the samples per iteration to balance variation while maintaining sufficient data to form robust clusters [26]. The number of iterations was set to 1000 to create stable cluster labels. Pearson’s correlation was chosen as the distance function, given the continuous and normally distributed data. Complete linkage was selected to measure the linear relationship of genes across samples and to form compact, well-separated clusters [27]. The optimal number of clusters was determined using several clustering indices, including the Silhouette score, Davies-Bouldin index, gap statistic, and Calinski-Harabasz index [28–31].

#### Multi-Omics factor analysis

Multi-omics factor analysis (MOFA) is a Bayesian probabilistic framework that explains data variance by reducing it into latent factors [32]. The available clinical features for each dataset were incorporated as views in the model, with the resulting latent factors used as input for the k-means + + clustering algorithm.

#### Autoencoder

Autoencoders are neural networks that compress data into a lower-dimensional latent space, capturing essential information and modelling non-linear relationships. Performance is based on decoding the information to recreate the original input. The model was built in Python using TensorFlow [33]. In this model, Tanh and sigmoid activation functions were used in hidden and output layers, respectively, to normalize outputs for robust clustering. Ridge regularisation (factor 0.0001) was applied to encoder layers and Lasso regularisation (factor 0.001) to the bottleneck layer. An Adam optimiser managed noisy gene expression data, with binary cross-entropy as the loss function, appropriate for measuring the difference between output and predicted probability in models using the sigmoid function. The model trained with a batch size of 32 over 1,000 epochs, with shuffled samples and a 20% validation split to prevent overfitting. The selected number of latent features for the bottleneck layer minimised reconstruction error, which was then used as input for K-means + + clustering, effectively capturing variance from integrated gene expression and clinical data.

#### K-means ++

K-means is a clustering algorithm that iteratively assigns samples to the nearest centroid, recalculates centroids, and minimises the average distances between centroids and samples. K-means + + builds upon this by systematically determining the initial centroid to not introduce randomness, and therefore optimising the clustering performance [34]. The *kmeanspp* function from the *maotai* package in R was used to repeat the centroid initialisation 1000 times for a stable cluster [35]. The same clustering indices used for hierarchical clustering were used to evaluate clustering performance for both MOFA and Autoencoder with K-means++.

### Overlap analysis

The adjusted rand index was used to measure the composition of patients in each cluster across the three clustering methods. Only the groups of patients that were consistently clustered across all three methods were used for further analysis. The patients that did not exhibit similar clustering patterns across the methods were excluded from further analysis.

### Influential genes and pathway enrichment analysis

A DGEA was then performed on the overlapping patient clusters to identify which genes were influential to the cluster assignments. A pathway enrichment analysis using the *enrichR* package in R was performed on the significantly DEGs for each cluster, specifically looking at the GO Biological Process 2021 and KEGG Human 2021 pathway [36, 37].

### Immune Deconvolution

The *ImmuneDeconV* package in R is a collection of immune deconvolution algorithms that estimate the proportions of immune cells from gene expression data of bulk tissue samples [38]. Predefined reference profiles of immune cell types decompose the patient’s mixed gene signals into its individual components to quantify the cell type fractions. Cell-type Identification By Estimating Relative Subsets Of RNA Transcripts (CIBERSORT) uses a support vector regression to deconvolute the tumour-infiltrating leukocyte abundance from a signature matrix of 547 immune marker genes [39]. The microarray data was permuted 1000 times for statistical confidence, with quantile normalisation applied to account for biological variance. A pairwise Wilcoxon test adjusted with Benjamini-Hochberg procedure was performed to identify which cluster was creating the most variance in each immune cell abundance [20]. Tumour Immune Estimation Resource (TIMER) gives an insight into tumour-immune interactions using constrained least squares regression, with Liver Hepatocellular Carcinoma (LIHC) as the reference profile [40]. Estimating the Proportions of Immune and Cancer cells (EPIC) uses a weighted non-negative least squares regression approach to estimate the abundance of immune, stromal, and cancer cells [41].

## Results

### Outlier detection

**Fig. 3:**
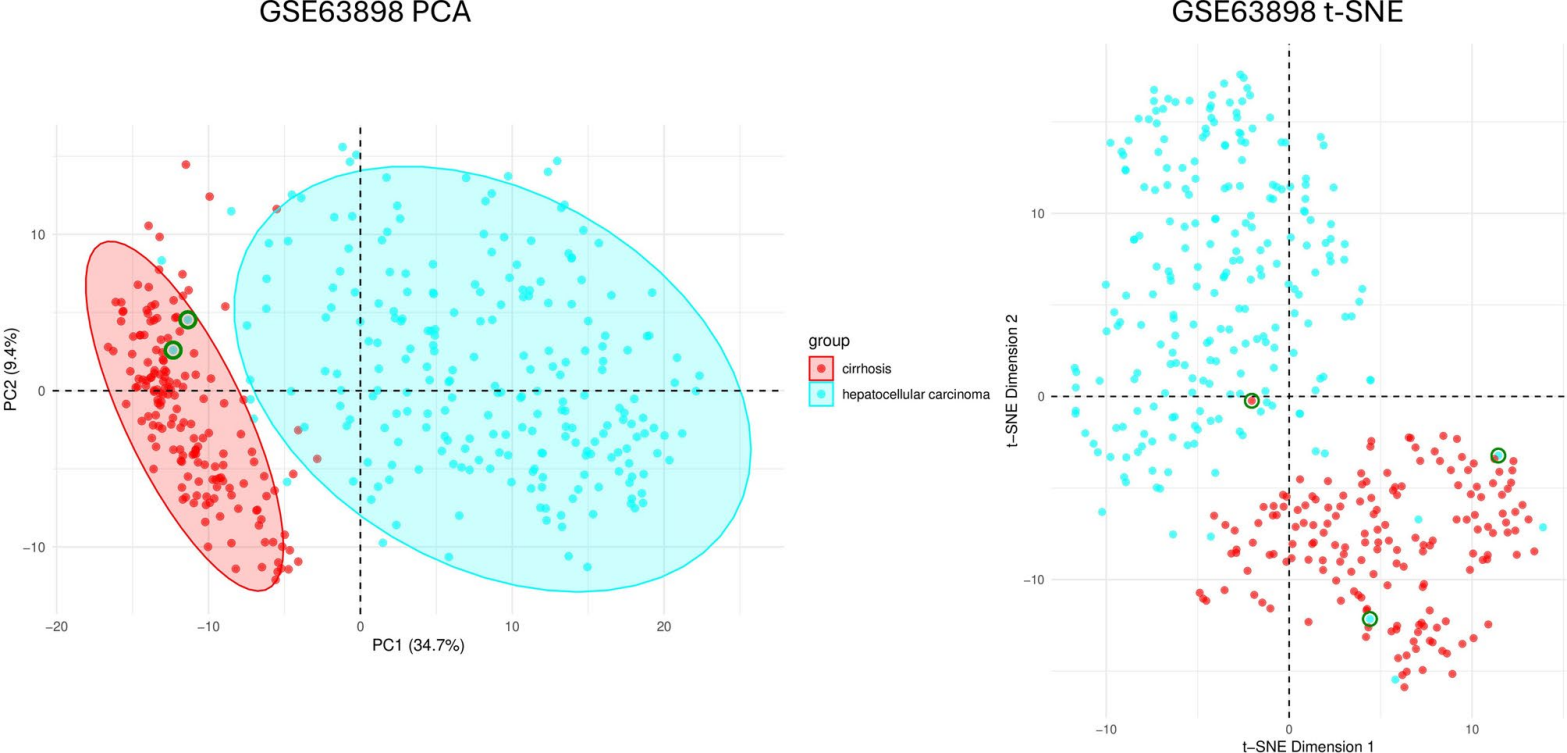
Principal component analysis (PCA) and t-distributed stochastic neighbour embedding (t-SNE) of discovery dataset, GSE63898. HCC Samples GSM1559895 and GSM1559920 and cirrhosis sample GSM1559861were identified as outliers in both plots and have been highlighted with a green circle. The other outlier samples were cross-referenced against each plot and were not found to be true outliers

Samples that were identified as outliers in PCA – those outside the 95% confidence ellipse - were compared against their location in the t-SNE plot (Fig. 3). If the sample was also anomalous, it was removed. Several samples appeared as outliers in one plot but clustered closely to others in the reference plot, demonstrating the importance of both global and local data representations. Two HCC samples, *GSM1559895* and *GSM1559920*, were identified and are marked with green circles in Fig. 3 – *GSM1559895* is partially obscured by a red cirrhosis sample in the t-SNE. One cirrhosis sample, *GSM1559861*, was also removed from downstream analysis. Two samples found to be outliers in the validation dataset, GSM363041 and GSM363088, were removed from further analysis.

### Feature selection

The primary dataset had 49,387 gene probes. After re-annotating the probes to the available HGNC name and cross-referencing with the genes present in the validation dataset, 11,015 genes remained. The DGEA left 121 genes meeting the cut-off of an absolute log-fold change greater than 2 and an adjusted p-value lower than 0.05.

These significant genes were inputted into the Boruta algorithm, which identified 46 genes as important for distinguishing between tumour and non-tumour samples. The area under the curve for the primary dataset was 0.998, with an accuracy of 0.983 (95% confidence interval: 0.9401, 0.998). The remaining genes were discarded.

### Clustering identifies distinct HCC patient subgroups

To identify subgroups of HCC patients with distinct molecular profiles, three unsupervised clustering methods were applied. Each approach revealed a consistent four-cluster structure, supporting the presence of biologically relevant heterogeneity.

#### Hierarchical clustering

**Fig. 4:**
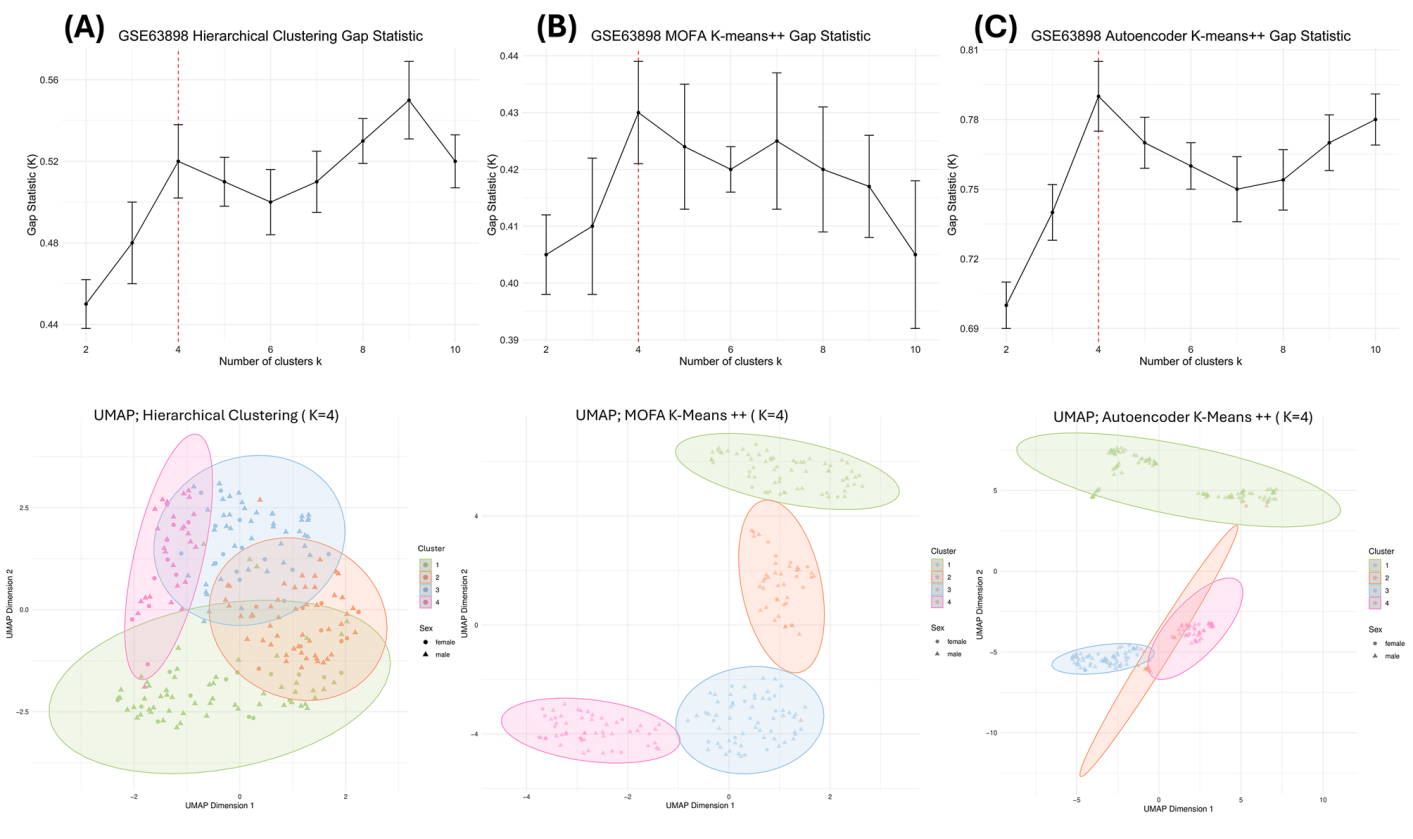
Gap statistic plot and Uniform Manifold Approximation and Projection (UMAP) of optimal clusters for the three unsupervised machine learning clustering methods: (**A**) hierarchical clustering, optimal number of clusters (K) is four (gap statistic = 0.52); (**B**) multi-omics factor analysis (MOFA) with K-means++, K is four (gap statistic = 0.43); (**C**) autoencoder with K-means++, K is four (gap statistic = 0.79). Error bars indicate standard deviation. The UMAPs show the distribution and shape of the identified clusters. The silhouette scores are 0.14, 0.17, and 0.52, respectively

By performing agglomerative hierarchical clustering 1000 times with 80% resampling on the Boruta-identified important gene expression data, the optimal number of patient clusters was found to be 4 (K = 4) (silhouette score = 0.14; Gap Statistic = 0.52, Davies-Bouldin index = 1.94, Calinski-Harabasz Index = 28.12) (Fig. 4, Supplementary Fig. 1). Although the gap statistic was slightly higher at K = 9 (0.55), K = 4 was selected to minimise the risk of overfitting and to ensure more interpretable and biologically meaningful subgrouping within the patient populationCluster 4 was found to have a clinically significant distribution of clinical features compared to all other clusters (Hotelling T-Squared test: test statistic = 10.73; p-value = 0.015). A univariate Student’s t-test revealed that the mean age in Cluster 4 was significantly lower than that of the remaining study population, at 61.63 years and 66.38 years, respectively (p-value = 0.016). Sex and tumour stage were not significantly different across all clusters.

#### MOFA with K-means++

**Fig. 5:**
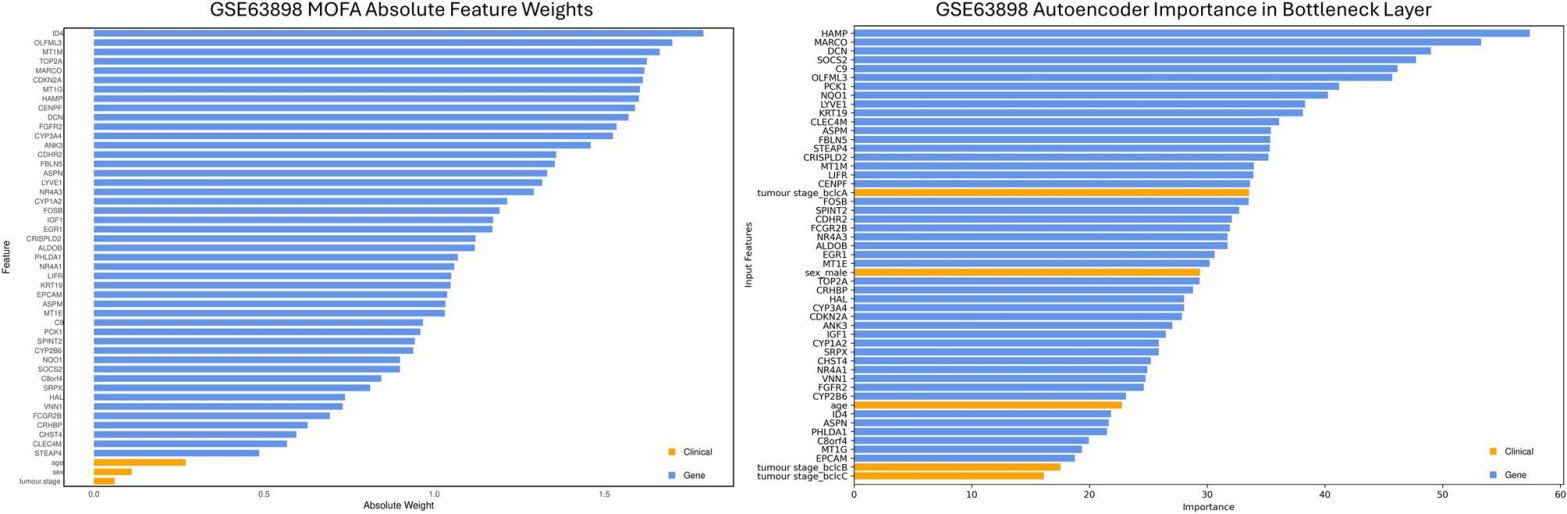
Bar plots of the absolute feature weights of the Multi-Omics Factor Analysis (MOFA), and the importance of features in the bottleneck layer from the autoencoder. The gene with the greatest feature weight across all latent factors for MOFA is ID4, with all clinical features having the least. HAMP has the greatest importance according to the autoencoder, with clinical features again having the least importance, although being more spread out

For training the MOFA model, all view likelihoods were set to Gaussian, except for sex, which was set to Bernoulli as it is binary. The data variance was explained using six latent factors, with Factor 1 accounting for the greatest variance, particularly influenced by the *DCN* gene (Fig. 5). Across all factors, *ID4* has the greatest absolute weight, while the three clinical attributes had the lowest absolute weights. Among the clinical features, sex showed the most variance, followed by tumour stage and age.

The six output latent factors were used as input for K-means + + clustering, where the optimal number of patient clusters determined to be four (K = 4) based on the clustering indices (silhouette score = 0.17; Davies-Bouldin Index = 1.86; Gap Statistic = 0.43; Calinski-Harabasz Index = 37.17) (Fig. 4B, Supplementary Fig. 2). The Hotelling T-squared test indicated that Cluster 4 had significantly different clinical features (test statistic = 10.67; p-value = 0.016), and a two-sided Student’s t-test confirmed that the mean age of Cluster 4 (62.47 years) was significantly lower than that of the remaining study population (66.24 years) (p-value = 0.046).

#### Autoencoder with K-means++

22 latent features at the bottleneck layer yielded the lowest reconstruction error, with a mean squared error of 0.431, making it the optimal choice. *HAMP* was the most important feature in the bottleneck layer, whereas tumour stages B and C were the least important overall (Fig. 5). These latent features were used as input for the K-means + + algorithm.

Using the same parameters as for the MOFA latent factors clustering, the bottleneck latent features identified four optimal patient clusters (K = 4). The clustering quality was supported by a silhouette score of 0.52, a Davies-Bouldin Index of 1.33, a Gap Statistic of 0.79, and a Calinski-Harabasz Index of 154.15 (Fig. 4C, supplementary Fig. 3). Further analysis showed that Cluster 3 had a significantly lower mean age of 62.11 years compared to 66.37 years for the rest of the population. This difference was statistically significant, as confirmed by both the Hotelling t-squared test (test statistic = 12.099, p-value = 0.0085) and Student’s t-test (p-value = 0.02).

### Overlap analysis reveals conserved subgroups across methods

**Fig. 6:**
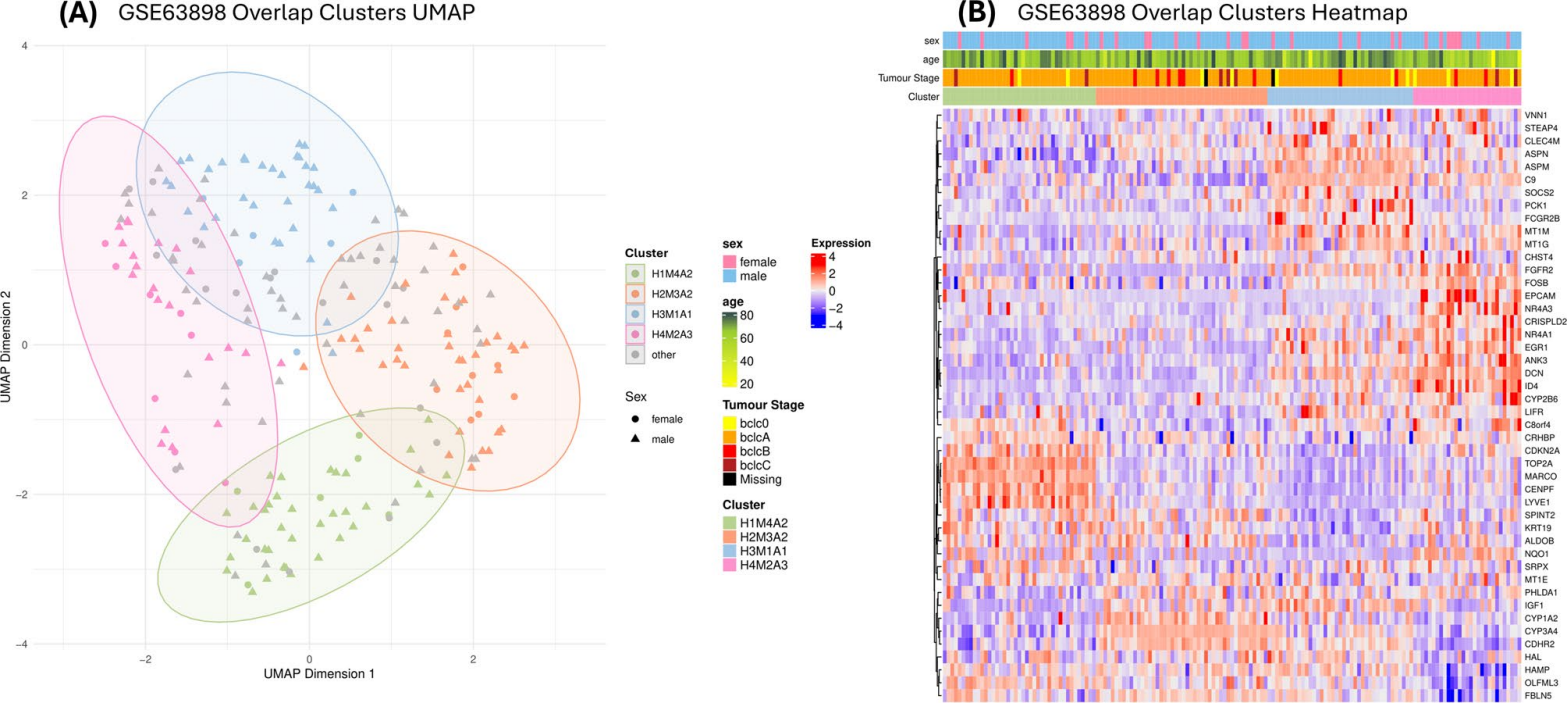
A Uniform Manifold Approximation and Projection (UMAP) and enriched heatmap of the overlap clusters. (**A**) UMAP of the four groups of patients conserved across all three clustering methods. The silhouette score for the clusters is 0.32. (**B**) Enriched heatmap of the differentially expressed genes across the overlap clusters. The clinical features sex, age, and tumour stage are given for each cluster. Age was the only clinical feature to be significantly different across clusters, with H4M2A3 (pink) having the youngest mean age

Comparing the clusters from each UML technique reduced method-specific bias and identified four consensus patient groups with consistent cluster membership across all methods, supporting the biological relevance of the subgroup structure.Four groups of patients were identified as conserved across all three clustering methods, each with an adjusted rand index greater than 0.4 (Fig. 6). The overlapping cluster names were derived from their original clusters; for example, H4M2A3 consisted of patients overlapping from hierarchical cluster 4, MOFA cluster 2, and Autoencoder cluster 3 – they are also referred to as overlap clusters Due to the size of Autoencoder cluster 2 (115 patients), there was no overlap between patients in H1M4A2 and H2M3A2. These overlapping groups varied in size, with H4M2A3 being the smallest (29 patients) and H2M3A2 the largest (45 patients), collectively representing 155 of the 225 HCC tumour patients. Patients not consistently re-clustered were excluded from further analysis.

As no specific algorithm was used to create the overlap cluster labels, only the silhouette score (0.32) and Davies-Bouldin index (0.58) were applicable as clustering validation metrics (Fig. 6). The enriched heatmap in Fig. 6 illustrates the DEGs across the four overlap clusters. H4M2A3 was the only cluster that exhibited a significantly different mean age, with an average age of 61.07 years compared to 66.18 years in the other clusters (Student’s t-test: p-value = 0.049). This difference was further supported by the Hotelling T-squared test statistic of 10.23 and a p-value of 0.019.

### Subgroup-Specific influential genes suggest distinct biological functions

To understand the consistent grouping of patients and which genes influenced stratification, differential gene expression analysis was performed by comparing each cluster against the others, excluding patients not retained in the overlap analysis. Pathway enrichment analysis revealed distinct biological processes potentially driving tumour behaviour in each group. This analysis identified 44 genes as significantly differentially expressed across the clusters, which were further examined through pathway enrichment analysis using GO Biological Process 2021 and KEGG Human 2021 pathways.

In Cluster H1M4A2, 22 genes were differentially expressed, with *MARCO* and *FCGR2B* associated with the KEGG “Phagosome” pathway (adj. *p* = 0.036) and GO terms “plasma membrane invagination” (adj. *p* = 0.026) and “phagocytosis, engulfment” (adj. *p* = 0.033), suggesting increased macrophage or monocyte activity. *IGF1* was downregulated and linked to several pathways, most notably the “PI3K-Akt signaling pathway” (adj. *p* = 0.043) - this may reflect impaired growth signalling, potentially influencing tumour proliferation [42].

Cluster H2M3A2 showed significant upregulation of *CYP1A2* and *CYP3A4*, associated with the KEGG “Metabolism of xenobiotics by cytochrome P450” (adj. *p* = 0.04) and “Linoleic acid metabolism” (adj. *p* = 0.02), possibly indicating a role for altered detoxification pathways or drug resistance mechanisms [43].

H3M1A1 had no significant KEGG pathways, but “Regulation of Immune Effector Process” and “Regulation of Humoral Immune Response” were both significantly associated due to the upregulation of *C9* and *FCGR2B* (adj. *p* = 0.03; 0.031).

The KEGG pathway “TGF-beta signaling pathway” is significantly associated with the cluster H4M2A3 due to the significantly upregulated *DCN* gene (adj. *P* = 0.04). The pathway is a known mediator of fibrosis and suppressor of vascular invasion via cancer-associated fibroblasts (CAFs), potentially providing this subgroup with a more immunoprotected microenvironment [5].*CYP2B6*, *CYP1A2*, and *CYP3A4* were associated with the “Chemical carcinogenesis” pathway. *EGR1*, also upregulated, was involved in multiple pathways, including “Regulation of hormone biosynthetic process” (adj. *p* = 0.043) and “PI3K-Akt signaling” (with *NR4A1*,* PCK1*,* FGFR2*) as well as “Linoleic acid metabolism” and “Metabolism of xenobiotics by cytochrome P450.”

### Immune Deconvolution reveals divergence across subgroups

To further characterise the TME of each patient subgroup, we performed immune deconvolution using three algorithms. This allowed us to estimate immune cell composition and assess whether immune infiltration patterns differed between clusters. The GSE63898 dataset was successfully deconvoluted using CIBERSORT, TIMER, and EPIC. CIBERSORT significantly deconvoluted all samples across clusters, while TIMER was unable to deconvolute 52 samples, likely due to variability in gene expression variability introducing inconsistent patterns that the algorithm cannot parse.

**Fig. 7:**
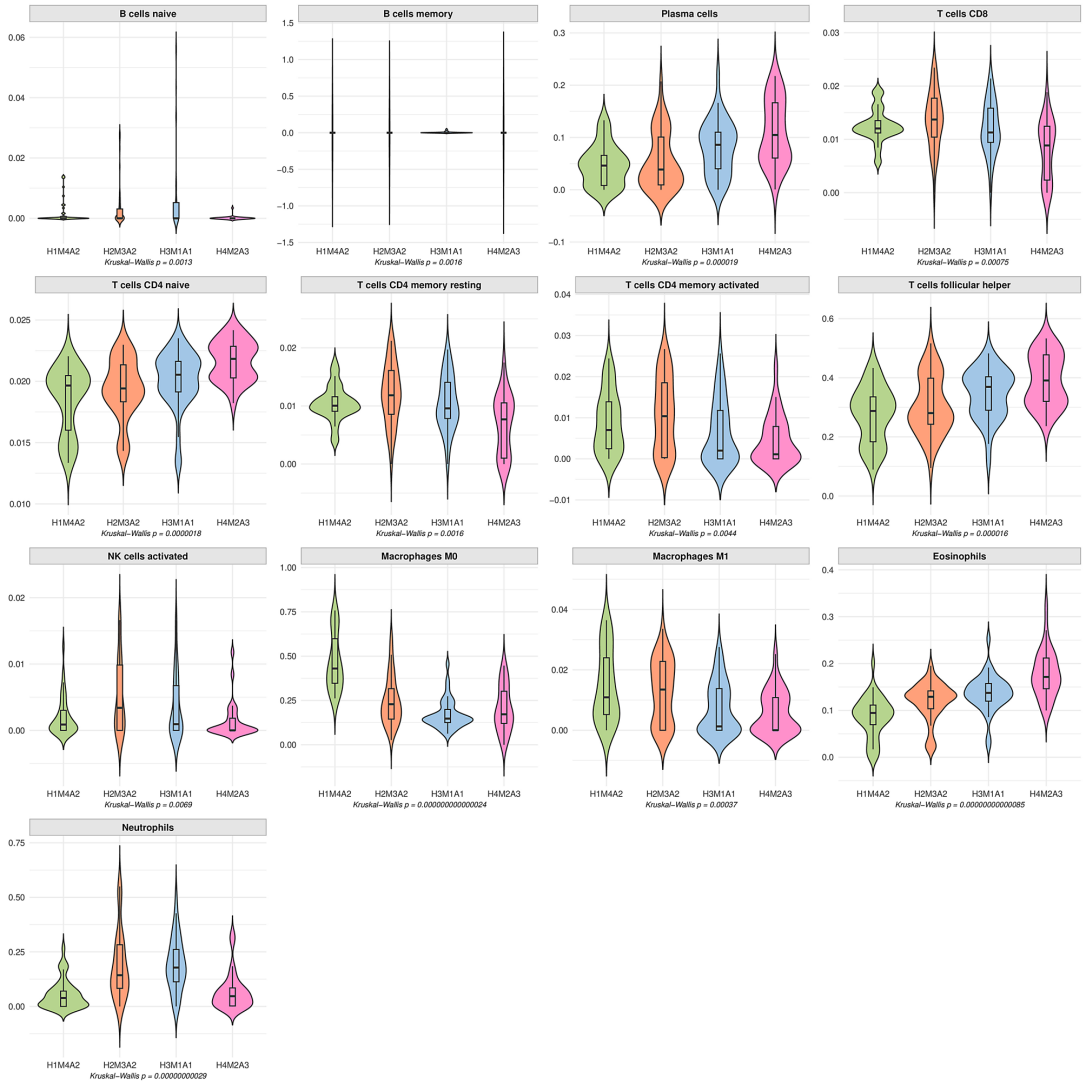
Violin plots of estimated immune cell abundance from CIBERSORT, the immune deconvolution algorithm. Kruskal-Wallis tests show the clusters have significantly different cell proportions across all clusters

Among the 22 immune cell types analysed by CIBERSORT, 13 showed significantly different abundances across patient clusters (Fig. 7), with diverse proportions observed, such as in eosinophils and neutrophils. In contrast, EPIC and TIMER showed fewer significant differences, with EPIC showing significance only in cancer-associated fibroblasts and macrophages, and TIMER in T cell CD4+, neutrophils, macrophages, and myeloid dendritic cells.

There was a disagreement between methods for T cells CD8 + and B cells: CIBERSORT found significant differences (*p* < 0.01 for CD8 + T cells, *p* = 0.013 for B cells), while TIMER did not estimate these abundances, and EPIC reported non-significant p-values (0.28 and 0.63, respectively). Macrophage abundance was significant in all deconvolution methods.

Pairwise Wilcoxon rank-sum tests indicated that H4M2A3 was significantly different from other clusters based on CIBERSORT results; however, these differences lost significance after adjusting for multiple testing using the Benjamini-Hochberg procedure. H4M2A3 was again identified as significantly different using TIMER-estimated abundances, with p-values of 0.028 compared to H1M4A2, 0.044 compared to H2M3A2, and 0.01 compared to H3M1A1. Although EPIC found two significant immune abundances, it did not detect any statistical differences between the groups. The significant differences in immune cell types detected by CIBERSORT - specifically eosinophils, neutrophils, and macrophages – supports the idea that immune heterogeneity contributes to the between-patient biological differences.

### External validation supports robustness of patient subgroups

The same methods of outlier detection, subtype stratification, overlap analysis, influential gene identification, and immune deconvolution was performed on the validation dataset GSE14520.

For outlier detection, GSM363041 and GSM363088 were removed due to being identified as outliers in both PCA and t-SNE analyses. For feature selection, the Boruta algorithm identified 83 genes as important for distinguishing between HCC and adjacent non-tumour samples.

#### Subtype stratification

MOFA and Autoencoder-based clustering both identified four optimal clusters (silhouette scores 0.25 and 0.64, respectively), while Hierarchical clustering suggested five clusters (silhouette score 0.33).

In MOFA, AFP serum had the highest absolute weight among all features, including genes, while the clinical features HBV status, sex, cirrhosis status, tumour size, and age were deemed the least important. Autoencoder identified *ESR1* as the most significant feature in the bottleneck layer, with CLIP stage V ranking second. Interestingly, unlike MOFA and the primary dataset, Autoencoder showed clinical features evenly distributed across the features, rather than concentrated at the bottom.

#### Overlap analysis

The overlap of patients across clustering methods in the validation dataset was much lower compared to the primary dataset. Only three clusters—H1M3A4, H2M2A2, and H4M4A3—had adjusted Rand indices greater than 0.2 so were retained for further analysis. These clusters included 57 out of the 211 patients used for clustering.

#### Influential genes

Total 58 genes were identified as significant for patient stratification, 13 of which overlapped with the influential genes from the primary dataset. A Fisher’s exact test indicated that this overlap was highly unlikely to have occurred by chance, with an odds ratio of 61.03 (p-value < 0.01).

#### Immune Deconvolution

Due to small cluster sizes, TIMER and EPIC could not be performed. Merging of relatively similar clusters was considered but ultimately deemed unsuitable as combining clusters would risk obscuring meaningful heterogeneity in tumour microenvironment profiles and potentially dilute subtype-specific immune signatures. CIBERSORT successfully deconvoluted immune cell abundances for all available patients. Although immune cell abundances were estimated for the three clusters, none showed statistically significant differences across clusters, with macrophage M0 and M2 being the closest at a p-value of 0.073. Using the Wilcoxon Rank-sum tests, the variance of each cluster was compared, and cluster H2M2A2 had the most amount of variance but was not significant.

#### Survival analysis

**Fig. 8:**
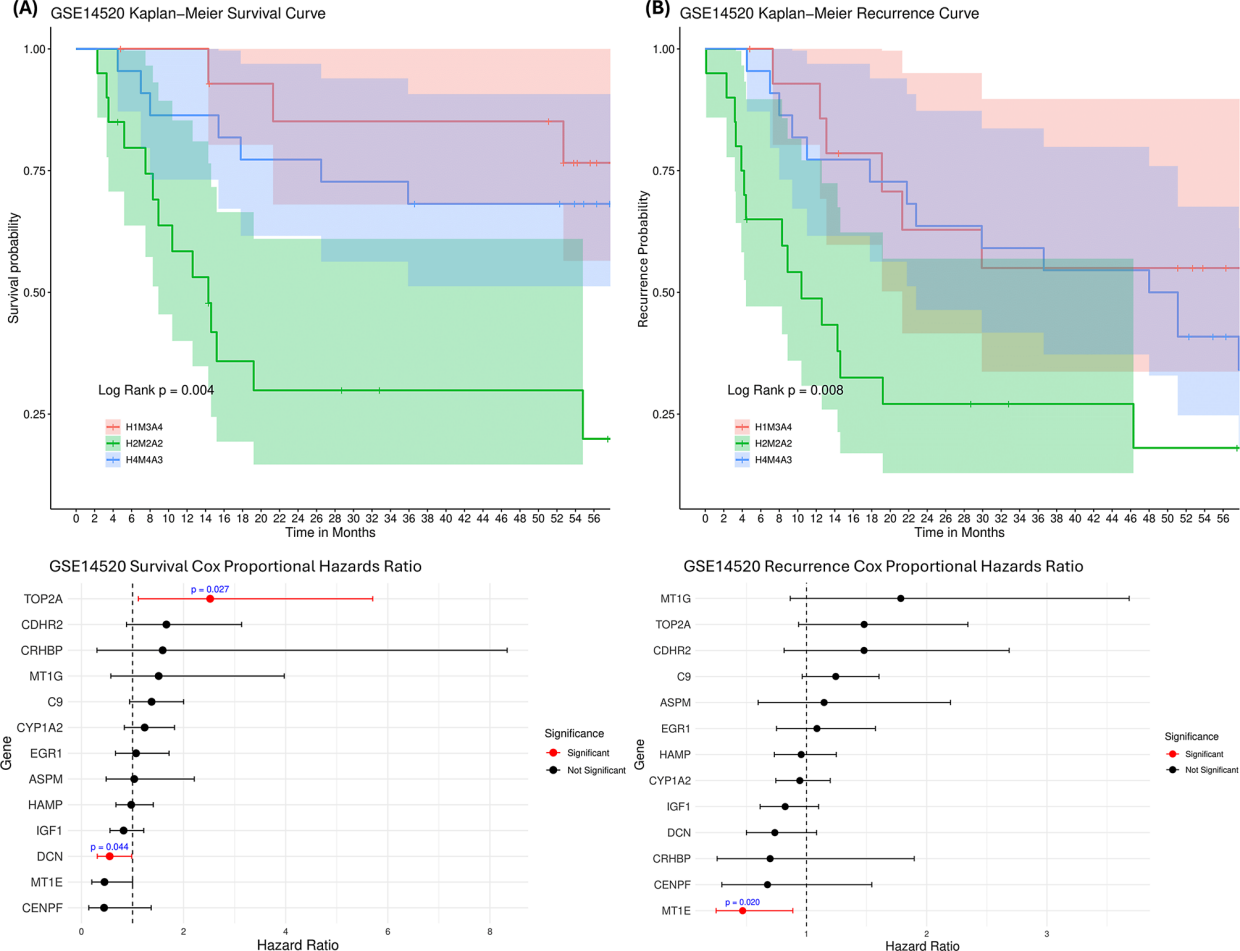
Kaplan-Meier survival and recurrence curves of validation (GSE15420) dataset, and Cox Proportional Hazards Ratios for survival and recurrence. (**A**) Kaplan-Meier and Cox proportional hazard ratios of hepatocellular carcinoma (HCC) survival. (**B**) Kaplan-Meier and Cox proportional hazard ratios of HCC recurrence. Error bars indicate standard deviation. H2M2A2 had the worst survival and HCC recurrence after 56 months of all clusters. TOP2A, DCN, and MT1E had significant hazards ratios at 2.52, 0.55, and 0.45, respectively. (p-value = 0.027; 0.044; 0.02)

the validation dataset included survival and recurrence data. Kaplan-Meier analysis showed significant survival differences between clusters (log-rank *p* = 0.004) (Fig. 8). Cox proportional hazards identified *TOP2A* as detrimental and *DCN* as protective for survival, with *MT1E* and *C9* showing borderline significance (*p* = 0.05 and 0.09). Cluster H2M2A2 had the worst survival, with 14 of 20 patients dying after 56 months, compared to 3 of 15 in H1M3A4. The recurrence pattern was similar, with H2M2A2 showing the most HCC recurrences (15 patients), 14 of whom also died. *MT1E* was the only gene with a significant protective effect against recurrence. The significant survival differences between clusters indicate that the molecular subgroups identified by UML are clinically relevant and may inform risk stratification.

## Discussion

This study utilised the unsupervised machine learning methods hierarchical clustering, MOFA with K-means++, and autoencoder with K-means++, to stratify patients and explore the heterogeneity of hepatocellular carcinoma as it relates to the tumour microenvironment. By integrating HCC gene expression data with clinical features, this novel approach aimed to provide a comprehensive understanding of the mechanisms driving cancer progression and identified potential biomarkers.

The BCLC system, while widely used for HCC prognosis and treatment stratification, does not account for the heterogeneity of the TME, which plays a crucial role in disease progression and therapeutic response [44]. As a result, it struggles to accurately predict the diverse treatment outcomes observed in HCC patients. To explore this limitation, we employed UML clustering techniques to analyse TME heterogeneity, aiming to improve prognosis and recurrence predictions beyond what the traditional BCLC classification can achieve. These clustering methods were chosen for their complementary strengths: hierarchical clustering identifies nested patterns within the data, MOFA integrates structured and unstructured data to elucidate complex relationships between features, and autoencoders capture important non-linear relationships by compressing data into a lower-dimensional representation. The adaptability of each method to unseen data fortifies their combined effectiveness in capturing the heterogeneity of HCC. However, the clustering indices for each UML method were not ideal, and even with hyperparameter tuning, further optimisation was not possible. This reflects the challenges of handling high-dimensional data, which often resists forming compact clusters – not uncommon for microarray analyses. Hierarchical clustering and MOFA with K-means + + produced relatively low scores (0.14 and 0.17, respectively), highlighting the difficulty in achieving clear separability within noisy data. In contrast, the autoencoder with K-means + + approach yielded a notably higher silhouette score (0.52), demonstrating better-defined clusters and greater internal consistency. The use of multiple clustering techniques gave methodological validation to the patient clusters identified in the overlap analysis in a context where clear boundaries are inherently difficult to define.Linking the TME to each cluster highlights the diverse nature of HCC, explaining why a “one-size-fits-all” approach to treatment is ineffectual.

This study gives an insight into the varied treatment responses observed in HCC patients, which may be attributed to differences in the tolerogenic nature of the TME and the resulting immune cell abundances. For instance, the mean proportion of eosinophils and plasma cells in the H4M2A3 cluster was nearly double that in H1M4A2 (Fig. 7). However, the reasons for the variations in the TME across identified clusters remain unclear. The significant variation in age across subtypes provides some explanation, as age at presentation of HCC has been linked to genetic variations, such as *UGT2B28*, suggesting that the genes identified in this study may serve as potential biomarkers for predicting the age of onset in HCC patients [45]. Discrepancies observed between the deconvolution algorithms likely stem from both methodological and biological factors, with stromal contamination—particularly from CAFs —emerging as a key contributor. Each method employs distinct reference gene signatures and regression models which can lead to divergent sensitivity in identifying immune and stromal components [39–41]. CIBERSORT detected a broader range of immune subsets, whereas EPIC primarily highlighted stromal components such as fibroblasts and macrophages. Liver tumours are characteristically fibrotic, with extensive stromal content driven by the accumulation of extracellular matrix and activated fibroblasts. In such settings, the abundance of stromal transcripts can dilute or obscure the detection of immune-related signals, particularly in microarray-based bulk tissue data where spatial context is lost. The presence and spatial distribution of specific fibroblast subsets can significantly alter the immune landscape within tumours, potentially leading to discrepancies in algorithmic estimations of immune cell populations [46]. Recent spatial multi-omics research has demonstrated that CAFs in HCC not only contribute significantly to the stromal signature but are also associated with cancer stemness and immune modulation, influencing tumour progression and therapeutic resistance [47]. Their presence may therefore skew immune deconvolution outputs, with algorithms like EPIC—which model stromal and cancer cells explicitly—being more responsive to their abundance, while others like CIBERSORT may underrepresent or misattribute signals in the presence of dominant stromal expression.

Thirteen genes were identified as influential in subtyping patients in both the primary and validation datasets, with three—*TOP2A*, *DCN*, and *MT1E*—showing significant associations with survival and recurrence in HCC (Fig. 8). The established roles of these genes in HCC further validate the findings of this study. *DCN*, or Decorin, an integral proteoglycan in the extracellular matrix, was consistently significant across both datasets and demonstrated a significant hazard ratio associated with improved survival in HCC patients. Known for its protective role as a tumour suppressor in HCC, gene enrichment analysis confirmed its involvement in the TGF-beta signalling pathway, a key mechanism whose aberration contributes to HCC malignance [48, 49]. Upregulation of *DCN* mitigates the pro-tumorigenic microenvironment by promoting the infiltration and activation of leukocytes, such as cytokines and chemokines, and creating a pro-inflammatory state by encouraging macrophage polarisation to the M1 phenotype [50]. *DCN* is secreted by cancer-associated fibroblasts [51], an immune cell type found to be abundant across all four clusters, further establishing its role in modulating the TME [6, 52]. These findings underscore the potential of *DCN* as a therapeutic target in HCC and suggest that its influence on the TME may be an unexplored factor in improving patient prognosis and survival rates, warranting further exploration. The limitations of using immune deconvolution algorithms are its inability to handle small sample sizes and unsuccessful deconvolution if the samples’ variance is too great. The implications of this are true biological outliers are not included in the analysis and the estimated cell proportion does not reflect the aberrant TME.

Performing overlap analysis across three UML clustering methods reinforces the robustness of patient stratification by identifying consistently recurring patterns, thereby mitigating the risk of overfitting inherent to individual clustering approaches. The integration of the clustering results ensures that identified subtypes reflect biologically meaningful heterogeneity rather than method-specific biases. Despite the study’s robust methodology, the validation results were unable to align with the findings from the primary dataset, highlighting a potential limitation in capturing the complete picture of HCC heterogeneity. However, it is important to note that these differences do not necessarily undermine the significance of the findings. Gao et al. (2020) identified four subclasses of the TME using a support-vector machine and highlighted the pivotal role of stromal cells in driving clinical outcomes and treatment responses to sorafenib [53]. This suggests that the immune microenvironment and TME complexity are influenced by more than just the gene expression profiles captured in this study. The primary dataset included patients from Barcelona, Spain, and Milan, Italy, whereas the validation dataset included patients from Shanghai, China. The lack of significant immune cell differences in the validation dataset underscores the possibility that other factors—such as stromal cell interactions, regional genetic variations, and environmental influences—might also play crucial roles in shaping the TME. Additionally, although statistical analysis can reveal significant associations between gene expression and the TME, it does not establish a direct causal relationship. Biological interactions are complex and influenced by multiple factors, requiring further experimental validation to confirm whether observed associations reflect underlying mechanistic pathways. Therefore, while the validation did not fully replicate the primary findings, it underscores the multifaceted nature of HCC and the need for further research to explore these additional layers of complexity.

Future research could benefit from integrating additional computational approaches to further refine our understanding of tumour heterogeneity and immune cell dynamics. Recent advances in multi-omics integration and tumour evolutionary modelling have demonstrated their potential to uncover complex relationships between genomic alterations and the TME [54]. Expanding our framework to incorporate such methodologies—particularly through the integration of whole-exome sequencing and broader transcriptomic datasets—may enhance the precision of patient stratification and improve our characterisation of immune interactions [55]. As tumour evolution and immune responses play critical roles in HCC progression, targeting these advanced analytical techniques could provide deeper insights into treatment responses and disease outcomes, offering a promising avenue for future investigation.

## Conclusions

The discovery of the 13 conserved genes across datasets is an important step toward understanding the genetic mechanisms of HCC and its variations. This could lead to the identification of more reliable biomarkers and therapeutic targets. The lack of significant immune cell differences in the validation dataset underscores the complexity of HCC, suggesting that the immune microenvironment is influenced by more than just gene expression, but also by factors not captured in the validation dataset.

The integration of multi-omics data would be the next logical step in expanding on the TME of HCC – specifically the integration of immunohistochemistry and tumour microbiome data. UML should again be employed to uncover the hidden interactions between features and elucidate the dependencies that promote hepatocarcinogenesis, with the identified cluster labels used in conjunction with a nomogram to predict patient prognosis based on immune cell abundance. Additionally, incorporating methodologies that account for tumour clonal evolution and broader transcriptomic profiling may provide a more comprehensive view of the tumour microenvironment, further refining patient stratification and treatment strategies.

## Electronic supplementary material

Below is the link to the electronic supplementary material.

Supplementary Material 1: Supplementary Figure 1: Clustering validation metrics for agglomerative hierarchical clustering of the GSE63898 dataset. (A) Silhouette score, (B) Davies-Bouldin index, and (C) Calinski-Harabasz index are shown for cluster numbers K=2 to K=10. Error bars represent variability based on repeated clustering with subsampling.

Supplementary Material 2: Supplementary Figure 2: Clustering validation metrics for MOFA-based clustering using K-means++ on the GSE63898 dataset. (A) Silhouette score, (B) Davies-Bouldin index, and (C) Calinski-Harabasz index are shown for cluster numbers K=2 to K=10. Error bars represent variability based on repeated clustering with subsampling.

Supplementary Material 3: Supplementary Figure 3: Clustering validation metrics for Autoencoder-based K-means++ clustering on the GSE63898 dataset. (A) Silhouette score, (B) Davies-Bouldin index, and (C) Calinski-Harabasz index are shown for cluster numbers K=2 to K=10. Error bars represent variability based on repeated clustering with subsampling.

## Data Availability

The discovery and validation datasets are openly available in the Gene Expression Omnibus at https://www.ncbi.nlm.nih.gov/geo/, accession numbers [GSE63898] and [GSE14520].

## Abbreviations

AFP: α-fetoprotein
BCLC: Barcelona clinic liver cancer
CAF: Cancer-associated fibroblast
CIBERSORT: Cell-type identification by estimating relative subsets of RNA transcripts
DEG: Differentially expressed genes
DGEA: Differential gene expression analysis
EPIC: Estimating the proportions of immune and cancer cells
HCC: Hepatocellular carcinoma
MOFA: Multi-omics factor analysis
PCA: Principal component analysis
RF: Random forest
TIMER: Tumor immune estimation resource
TME: Tumor microenvironment
t-SNE: t-distributed stochastic neighbour embedding
UML: Unsupervised machine learning
