# Supplementary figures and images for: Unsupervised machine learning-based stratification and immune deconvolution of liver hepatocellular carcinoma
Source: BMC Cancer. 2025 May 10;25:853. doi: 10.1186/s12885-025-14242-5 (PMC12066050; doi:10.1186/s12885-025-14242-5)

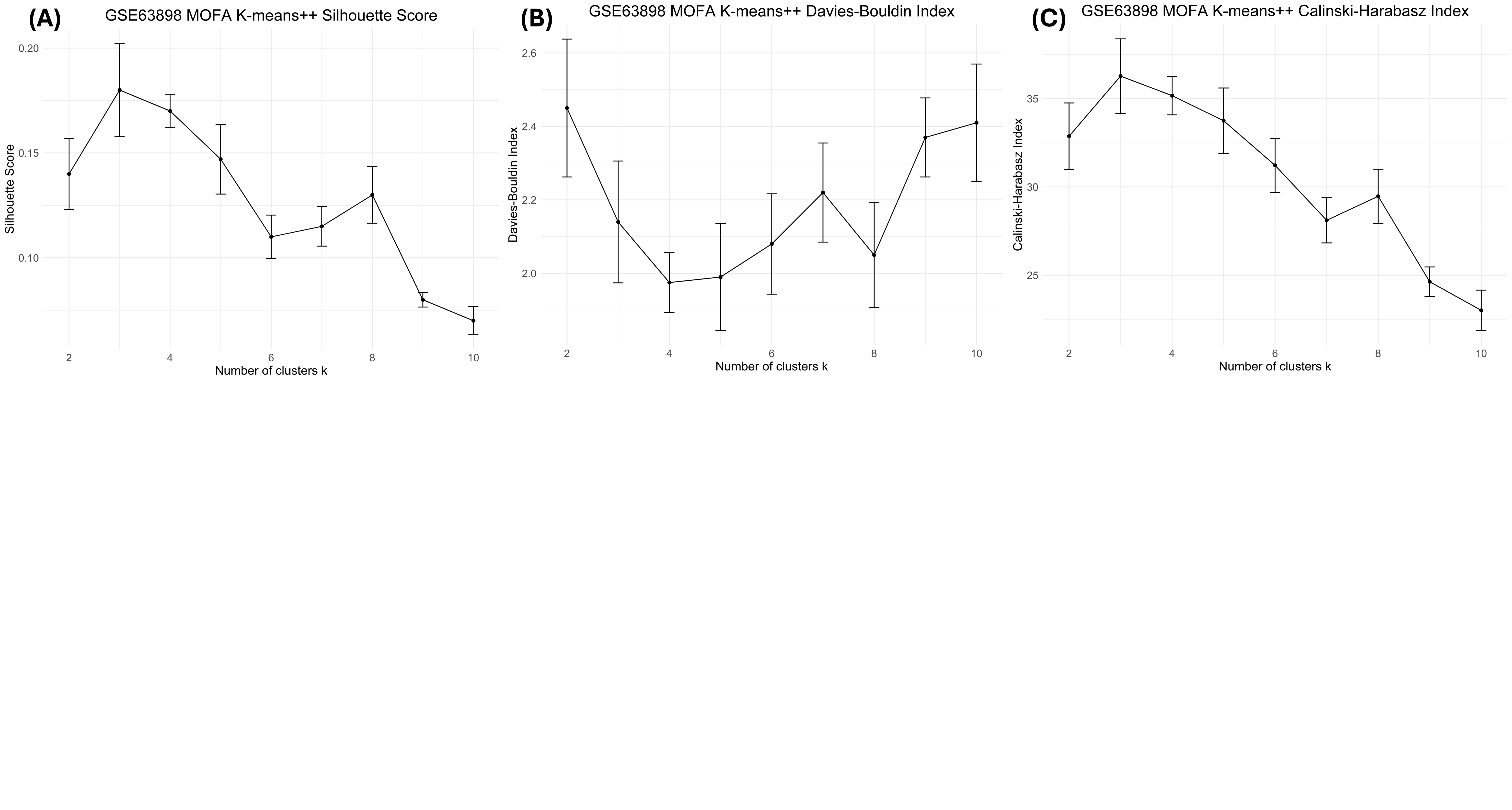

Supplement: Supplementary file 1 — Supplementary Material 1: Supplementary Figure 1: Clustering validation metrics for agglomerative hierarchical clustering of the GSE63898 dataset. (A) Silhouette score, (B) Davies-Bouldin index, and (C) Calinski-Harabasz index are shown for cluster numbers K=2 to K=10. Error bars represent variability based on repeated clustering with subsampling. [file 12885_2025_14242_MOESM1_ESM.png]

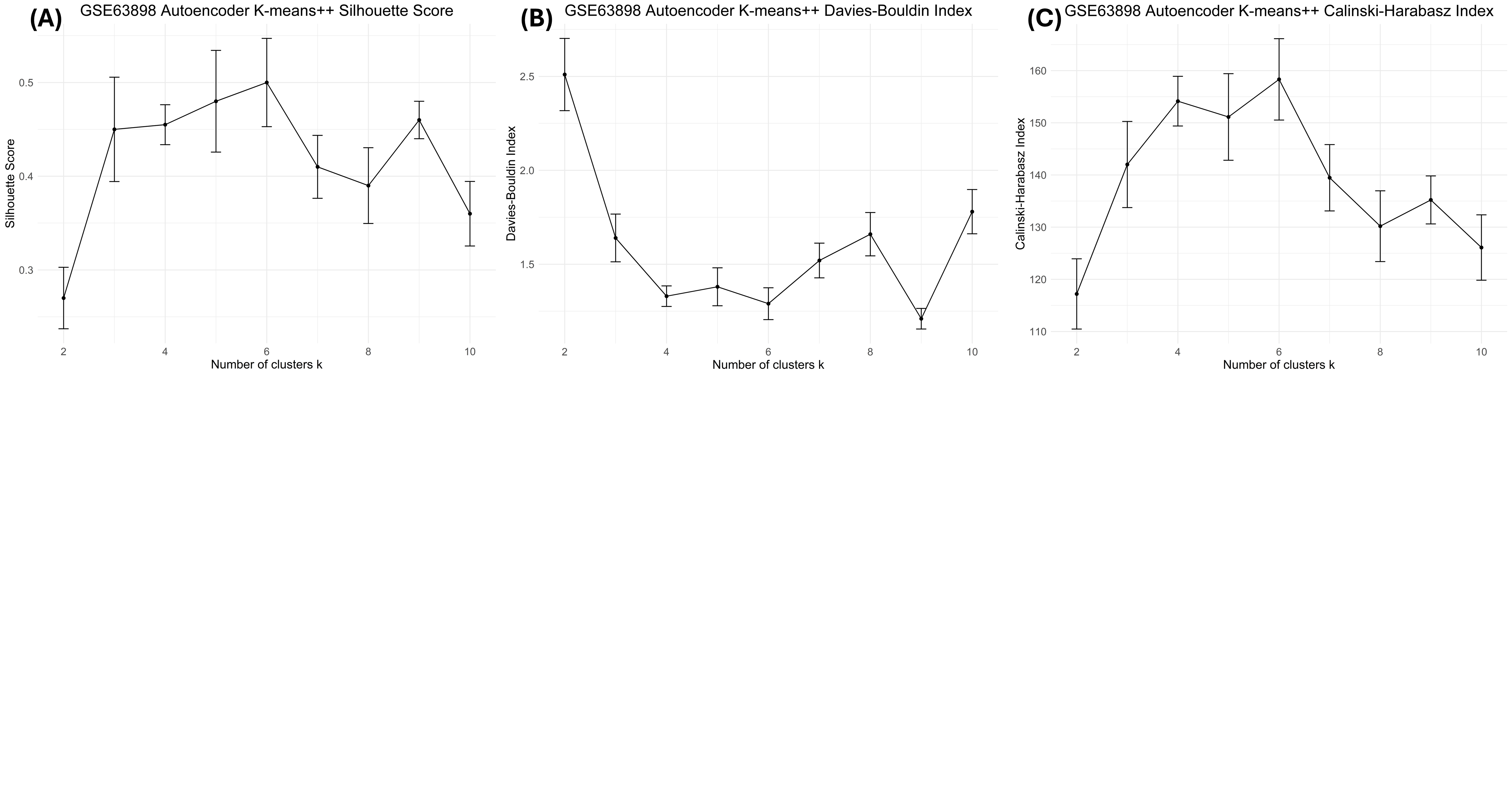

Supplement: Supplementary file 2 — Supplementary Material 2: Supplementary Figure 2: Clustering validation metrics for MOFA-based clustering using K-means++ on the GSE63898 dataset. (A) Silhouette score, (B) Davies-Bouldin index, and (C) Calinski-Harabasz index are shown for cluster numbers K=2 to K=10. Error bars represent variability based on repeated clustering with subsampling. [file 12885_2025_14242_MOESM2_ESM.png]

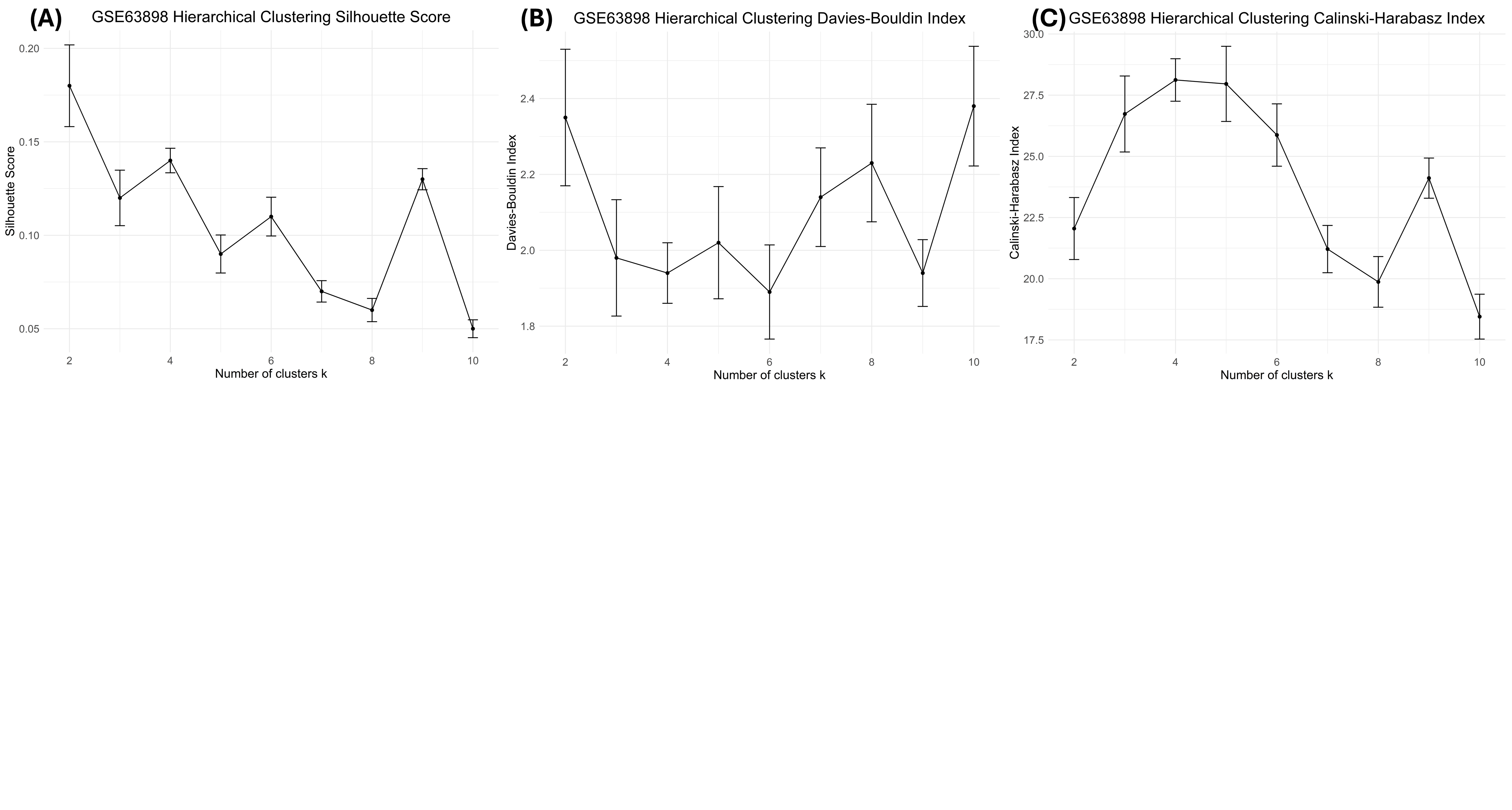

Supplement: Supplementary file 3 — Supplementary Material 3: Supplementary Figure 3: Clustering validation metrics for Autoencoder-based K-means++ clustering on the GSE63898 dataset. (A) Silhouette score, (B) Davies-Bouldin index, and (C) Calinski-Harabasz index are shown for cluster numbers K=2 to K=10. Error bars represent variability based on repeated clustering with subsampling. [file 12885_2025_14242_MOESM3_ESM.png]
